# High Mg effective incorporation in Al-rich Al_*x*_Ga_1 *- x*_N by periodic repetition of ultimate V/III ratio conditions

**DOI:** 10.1186/1556-276X-9-40

**Published:** 2014-01-21

**Authors:** Tongchang Zheng, Wei Lin, Duanjun Cai, Weihuang Yang, Wei Jiang, Hangyang Chen, Jinchai Li, Shuping Li, Junyong Kang

**Affiliations:** 1 Department of Physics, Fujian Key Laboratory of Semiconductor Materials and Applications, Xiamen University, Xiamen 361005, People’s Republic of China

**Keywords:** First-principles calculations, MOVPE, AlGaN epilayer, High Mg incorporation, Ultimate V/III ratio

## Abstract

According to first-principles calculations, the solubility of Mg as a substitute for Ga or Al in Al_*x*_Ga_1 *– x*_N bulk is limited by large, positive formation enthalpies. In contrast to the bulk case, the formation enthalpies become negative on Al_*x*_Ga_1 *– x*_N surface. In addition, the N-rich growth atmosphere can also be favorable to Mg incorporation on the surface by changing the chemical potentials. On the basis of these special features, we proposed a modified surface engineering technique that applies periodical interruptions under an ultimate V/III ratio condition (extremely N-rich), to enhance Mg effective incorporation. By optimizing the interruption conditions (2 nm interruption interval with 2 s interruption time), the enhancement ratio can be up to about 5 in the Al_0.99_Ga_0.01_N epilayer.

## Background

Al_*x*_Ga_1 *– x*_N alloys have attracted considerable attention in recent years because of their great potential for applications in UV and deep UV optoelectronic devices with spectral lengths as short as 200 nm [1]. Both high-quality *p*-type and *n*-type AlGaN epilayers are strongly demanded for electrical injection in constructing these short wavelength devices. However, similar to most wide bandgap semiconductors, AlGaN suffers from the ‘asymmetric doping’ limitation [2,3], i.e., doping AlGaN to form *n*-type layer is easy, but achieving *p*-type doping is difficult [4,5]. Although Mg is the most widely adopted *p-*type dopant for AlGaN, its doping efficiency is extremely low, particularly for high Al content Al_*x*_Ga_1 *– x*_N [6]. The low doping efficiency of Mg is mainly attributed to its limited solubility, high activation energy, and compensation effect with impurities or native donor defects [2,7]. In spite of the extensive efforts to improve the Mg activation efficiency [5,6,8,9], the bottleneck of low Mg solubility in GaN [10] and AlN [11] materials strongly restricts the overall *p*-type doping in AlGaN.

Regarding the dopant solubility issue, an extremely high carbon dopant concentration was shown to exist on the epitaxial surface of Si system [12]. This high concentration can be attributed to the surface enhancement effect caused by the partial release of atom mismatch strain. As the epitaxy continues, part of this high concentration dopant segregates to the new surface, and the residual components freezes into the host matrix [12] which corresponds to the final dopant concentration. In other words, the growing surface plays a critical role in determining dopant solubility. If the transient solubility on the growing front surface can be effectively enhanced, high dopant incorporation can be achieved. Theoretical simulations have recently predicted that a N-rich condition is beneficial for Mg incorporation in GaN and AlN [10,11]. However, high V/III ratio was determined to be unfavorable for high-quality Al_*x*_Ga_1 *– x*_N crystal growth [13-16]. Thus, the dilemma between maintaining high V/III ratio to promote Mg incorporation and maintaining low V/III ratio to ensure high crystal quality presents a long-standing challenge for deep UV optoelectronic devices.

In this work, we proposed a method to solve this V/III ratio dilemma by periodically interrupting the AlGaN growth (using usual V/III ratio as the AlGaN growth) and by shortly producing an ultimate V/III ratio condition (extremely N-rich). First-principles simulations were utilized to analyze the behavior of substituting Mg for Al and Ga in the bulk and on the surface of Al_*x*_Ga_1 – *x*_N under different growth atmospheres and to demonstrate the mechanism for the preferred Mg incorporation. On the basis of the analysis results, a modified surface engineering (MSE) technique that utilizes periodical interruptions under an extremely N-rich atmosphere was applied to enhance Mg effective incorporation by metalorganic vapor phase epitaxy (MOVPE). Significant Mg incorporation improvements in Al-rich Al_*x*_Ga_1 *– x*_N epilayer were achieved.

## Methods

The first-principles total energy calculations based on density functional theory were performed by using the Vienna *ab initio* simulation package [17]. Pseudopotentials were specified by the projector augmented wave [18,19] and by generalized gradient approximation [20]. Ga 3*d* electrons were treated as part of the valence band, and the plane wave cutoff energy was set at 520 eV. Geometry optimizations were performed until the total energy converged to 1 meV. For the bulk calculations, a 2 × 2 × 4 supercell containing 64 atoms [7] and a 5 × 5 × 3 Monkhorst-Pack grid [21] of *k*-points were used. All atoms were allowed to relax fully for energy minimization. For the surface calculations, we employed a 2 × 2 supercell with six Al_*x*_Ga_1 *– x*_N bilayers separated by a 13-Å wide vacuum region [22] and a 4 × 4 × 1 *k*-point mesh. The back side of the slab was saturated with hydrogen atoms of fractional charge. The three bottom Al_*x*_Ga_1 *– x*_N bilayers were fixed in the appropriate bulk-optimized configuration to simulate the growth surface, in which all the other layers was relaxed fully.

The Mg-doped Al_*x*_Ga_1 *– x*_N samples were grown on (0001) sapphire substrates via MOVPE. Trimethylgallium (TMGa), trimethylaluminum (TMAl), bis-cyclopentadienylmagnesium (Cp_2_Mg), and ammonia (NH_3_) were used as precursors, and H_2_ was used as carrier gas. Buffer layers with a 20-nm low temperature AlN nucleation layer, a 1-μm high temperature AlN layer, and a graded composition AlGaN layer have been used for initial growth on sapphire. The conventional method for fabricating Mg-doped Al_*x*_Ga_1 *– x*_N was conducted by retaining all the precursors during Mg-doped growth with usual V/III ratio. The MSE technique was implemented by periodically interrupting the conventional growth mode with closing the metal flows (TMAl, TMGa, and Cp_2_Mg) and continuously maintaining the NH_3_ flow to shortly produce an ultimate V/III ratio. The Mg and H concentrations were measured by using the Quad PHI 6600 secondary ion mass spectrometer (SIMS) system with depth resolution of approximately 2 nm, and Cs^+^ ion beams were used as primary ion sources.

## Results and discussion

**Figure 1:**
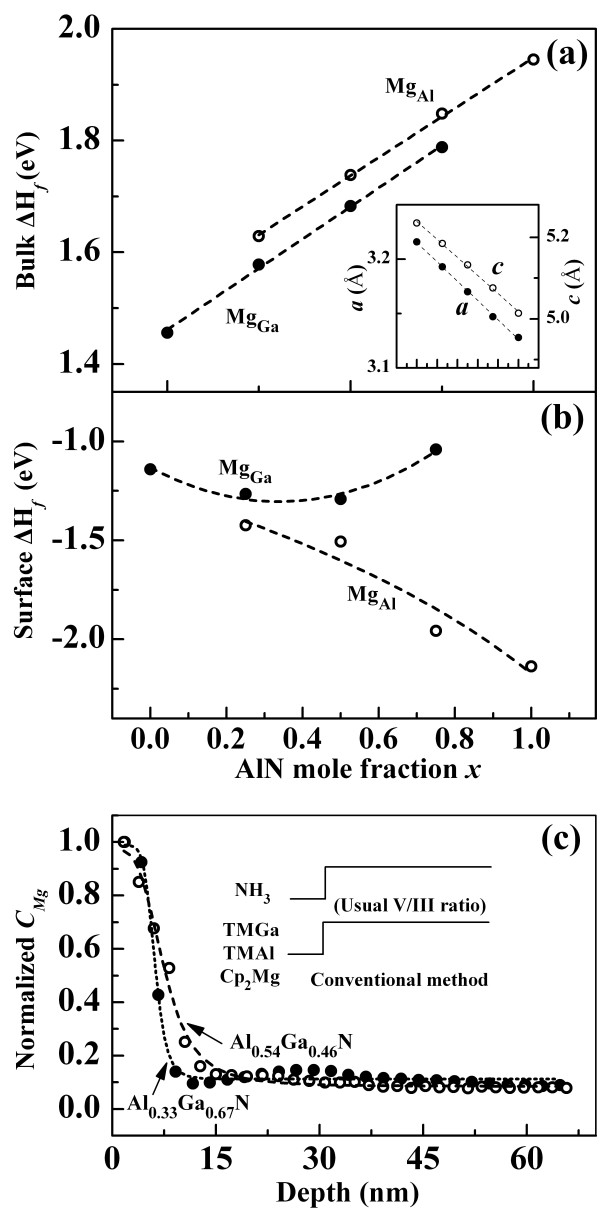
**Formation enthalpies of Mg**_**Ga**_**/Mg**_**Al**_** and normalized *C***_**Mg**_** cprofile of AlGaN films. (a)** In the bulk and **(b)** on the surface of Al_*x*_Ga_1 *-*__*x*_N as a function of Al content under N-rich condition. **(c)** Normalized *C*_Mg_ of Al_*x*_Ga_1 *-*__*x*_N (*x* = 0.33, 0.54) epilayers from the surface to bulk. The inset in (a) shows the calculated Al_*x*_Ga_1 *-*__*x*_N lattice constants *a* and *c* as a function of Al content. The inset in (c) illustrates the source supply sequence of the conventional method.

Considering that MOVPE growth is usually characterized by N-rich growth, we first discuss the formation enthalpies of neutral charge state Mg substituting for Al (Mg_Al_) and Ga (Mg_Ga_) in Al_*x*_Ga_1 *– x*_N bulk as a function of Al content under N-rich condition. The calculated results are shown in Figure 1a, wherein both the Mg_Al_ and Mg_Ga_ formation enthalpies are positive and large, thus indicating limited Mg solubility. The formation enthalpies of Mg_Al_ in AlN and Mg_Ga_ in GaN are comparable with previous results [10,11]. As the Al content in Al_*x*_Ga_1 *– x*_N increases, both the Mg_Al_ and Mg_Ga_ formation enthalpies monotonically increase. The formation enthalpy Δ*H*_*f*_ is closely related to the equilibrium Mg solubility *C*, which is given by [10]:

(1) $$C=N_{\mathrm{sites}}e^{-{\mathrm{\Delta H}}_{f}/k_{B}T}$$

where *N*_sites_ is the number of sites on which the dopant can be incorporated, *k*_*B*_ is the Boltzmann constant, and *T* denotes the temperature. Large formation enthalpy yields low dopant solubility. At the growth temperature (*T* = 1,000°C), the Mg solubility in bulk GaN is approximately 1.65 × 10^17^ cm^-3^. Considering that Δ*H*_*f*_ increases with increasing Al content, Al_*x*_Ga_1 *– x*_N experiences an aggravating Mg solubility limit. The Mg solubility limit may even decrease to approximately 2.32 × 10^16^ cm^-3^ in AlN (for *T* = 1,200°C). On the basis of this tendency, incorporating Mg becomes more difficult in Al-rich Al_*x*_Ga_1 *– x*_N. Notably, the formation enthalpy for Mg_Al_ is larger than that for Mg_Ga_ over the entire Al content range. This characteristic demonstrates that substituting Mg for Al is more energetically unfavorable than substituting Mg for Ga, which also explains the low Mg incorporation in Al-rich Al_*x*_Ga_1 *- x*_N. Such behavior of Mg is partly attributable to its larger covalent radius (1.36 Å) compared with those of Al (1.18 Å) and Ga (1.26 Å), as well as the compressive strain after Mg substitution [23,24]. As shown in the inset of Figure 1a, the Al_*x*_Ga_1 *– x*_N lattice constants *a* and *c* decrease as the Al content increases, thus making the mismatch strain caused by substituting Mg for Al or Ga atoms with smaller radii becomes more considerable.

Reducing the formation enthalpy is believed to be the key issue in solving the problem of Mg incorporation. The formation enthalpy is governed by two important factors, as given by [11]:

(2) $${\mathrm{\Delta H}}_{f}\left(\mathrm{neutral}\;\mathrm{state}\right)=\mathrm{\Delta E}+\Delta \mu .$$

Here, Δ*E = E*_Mg_ *- E*_host_, where *E*_Mg_ and *E*_host_ are the total energies of the supercell with and without Mg substitution; Δ*μ* = *μ*_Al/Ga_*– μ*_Mg_, where *μ*_*i*_ (*i* = Al, Ga, Mg) is the chemical potential. Δ*E* is mainly induced by the strain caused by the atom size mismatch. Consequently, larger atom size mismatch results in large Δ*E*, thus resulting in larger Δ*H*_*f*_ as mentioned above. The strain induced by the C-dopant into the Si matrix becomes smaller on the surface than in the bulk [12]. The question of whether the surface also plays a similar role in the Mg incorporation in Al_*x*_Ga_1 *– x*_N arises. To address this issue, we further investigated the formation enthalpies of Mg_Al_ and Mg_Ga_ on Al_*x*_Ga_1 *– x*_N surface, and the results are shown in Figure 1b. In contrast to the bulk case, both of the formation enthalpies in the surface are negative, suggesting favorable Mg substitution. The value of Mg_Al_ becomes lower than that of Mg_Ga_ and decreases as the Al content increases. These interesting reversed tendencies provide us a possible way to promote the Mg incorporation in Al-rich Al_*x*_Ga_1 *– x*_N by utilizing the surface effect. An epitaxy growth, e.g., MOVPE and molecular beam epitaxy systems, is conducted under an inherently non-equilibrium process with the surface transforming into a bulk [12]. Therefore, enhancing the Mg incorporation by using the surface effect should be practically feasible.

Two Mg-doped Al_*x*_Ga_1 *– x*_N (*x* = 0.33, 0.54) films were grown by MOVPE using the conventional method (the inset of Figure 1c) to validate the prediction of the surface effect on Mg incorporation. As shown in Figure 1c, the Mg concentration (*C*_Mg_) on the surface is about one order higher than that of in the bulk for both samples. Although *C*_Mg_ rapidly falls beneath the topmost surface (about 10 nm), *C*_Mg_ is still several orders higher than the theoretical prediction by Equation 1. This phenomenon can be understood in terms of the competition between the Mg incorporation enhancement on the growing surface due to the surface effect and the Mg segregation as the epitaxy continues. Simply, when the surface with the enhanced Mg solubility is covered by newly added layers during further growth, most of these Mg segregates to the new surface to regain equilibrium because the surface transforms into a bulk. Meanwhile, considerable part of these Mg is frozen in because of solidification. As a result, the *C*_Mg_ in the bulk is lower than that of in the final epilayer surface but is much higher than the equilibrium value of the ideal bulk. Considering this competition, Mg incorporation can be modified by balancing the surface time and solidification time.

As shown in Equation 2, the factor Δ*μ* also affects Mg incorporation. In principle, the chemical potential *μ* sensitively depends on the growth atmosphere (N-rich or Al/Ga-rich). The *μ* of a given species under equilibrium conditions is equal in all phases that are in contact [22]. Therefore, we can obtain

(3) $$\begin{array}{c} \mu_{\mathrm{Al}/\mathrm{Ga}}=\mu_{\mathrm{ANl}/\mathrm{GaN}}-\mu_{N}\\ \;=\mu_{\mathrm{Al}\left[\mathrm{bulk}\right]/\mathrm{Ga}\left[\mathrm{bulk}\right]}+\mu_{N_{2}}+\Delta H_{\mathrm{AlN}/\mathrm{GaN}}-\mu_{N}. \end{array}$$

In addition, *C*_Mg_ is limited by the formation of Mg_3_N_2_ to substitute Mg for Ga or Al as an acceptor [10]. This limitation meets the relation

(4) $$\mu_{\mathrm{Mg}}\leq 1/3\left(\mu_{Mg_{3}N_{2}}-2\mu_{N}\right).$$

**Figure 2:**
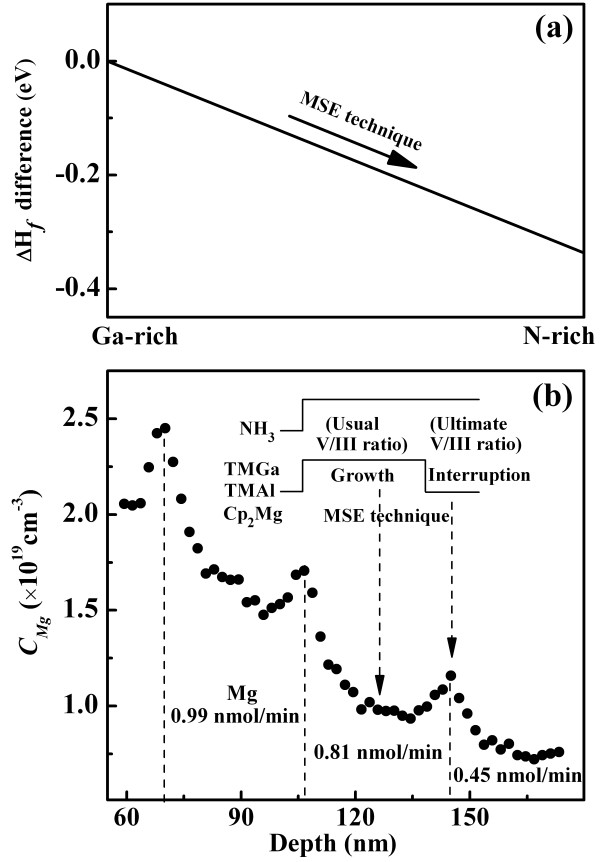
**Formation enthalpy difference of Mg**_**Ga**_**/Mg**_**Al**_** and *C***_**Mg**_** profile of Al**_**0.49**_**Ga**_**0.51**_**N film. (a)** Formation enthalpy difference of Mg_Ga_ and Mg_Al_ between Ga-rich and N-rich condition. **(b)***C*_Mg_ profile of Al_0.49_Ga_0.51_N film with three different Cp_2_Mg flows grown by the MSE technique. The inset in **(b)** illustrates the source supply sequence of the MSE technique, an ultimate V/III ratio condition is shortly produced during the interruption.

By substituting Equations 3 and 4 into Equation 2, we can obtain

(5) $$\Delta H_{f}\geq \mathrm{\Delta E}+\mu_{\mathrm{AIN}/\mathrm{GaN}}-1/3\mu_{Mg_{3}N_{2}}-1/3\mu_{N},$$

which, aside from Δ*E*, depends only on *μ*_*N*_, since the *μ*_AlN/GaN_ and $\mu_{Mg_{3}N_{2}}$are constants [25]. *μ*_N_ should be limited between *μ*_N_ (Al/Ga-rich) *≤ μ*_N_ *≤ μ*_N_ (N-rich) [11], namely, $\text{max}\left(\mu_{N_{2}}+{\Delta H}_{AIN},\;\mu_{N_{2}}+{\Delta H}_{GaN}\right)\leq \mu_{N}\leq \mu_{N_{2}}$, to drive the source materials to form Al_*x*_Ga_1 *– x*_N alloys instead of the undesirable phases (bulk Ga, Al, and N_2_). Our calculated ΔH_GaN_ value of -1.01 eV is higher than the ΔH_AlN_ value of -2.97 eV, which are consistent with the experimental values of -1.08 and -3.13 eV [25]. Therefore, as the growth condition varies from Ga-rich to N-rich conditions, *μ*_N_ changes from $\mu_{N_{2}}+{\Delta H}_{GaN}$ to $\mu_{N_{2}}$. Thus, Δ*H*_*f*_ varies over a range corresponding to 1/3Δ*H*_GaN_ of 0.337 eV, as shown in Figure 2a. This variation indicates that the N-rich growth atmosphere favor the Mg incorporation effectively in AlGaN. Generally, the N-rich condition is modulated by increasing the V/III ratio. However, for a fixed III flow, the Al_*x*_Ga_1 *– x*_N growth has an optimal V/III ratio for the best crystal quality [13-16]. Nonetheless, the max flow limitation of the MOVPE system does not allow the V flow to be increased infinitely. Accounting for these limitations, an inspiration can be obtained from Figure 1c, in which the protecting atmosphere with NH_3_ flow just provides an ultimate V/III ratio condition (extremely N-rich) for *C*_Mg_ enhancement when the epitaxy ends with the III flow becoming zero. Simultaneously, the stopped growth avoids the formation of low-quality Al_*x*_Ga_1 *– x*_N crystal. If this special condition is introduced as an intentional interruption during the continuous *p*-Al_*x*_Ga_1 *– x*_N growth, then the overall Mg incorporation could be improved.

To validate this hypothesis, a growth interruption experiment was designed, as shown schematically in the inset of Figure 2b. We closed the metal flows (TMAl, TMGa, and Cp_2_Mg flows) three times. In these three periods (35 nm thick), different Cp_2_Mg flows (0.45, 0.81, and 0.99 nmol/min) were applied to investigate the interruption effect systematically. Figure 2b shows the SIMS *C*_Mg_ profile of Al_0.49_Ga_0.51_N film across three periods. Obviously, *C*_Mg_ exhibits three distinct peaks at the interruptions, and the profile of each interruption is similar to that of the final epilayer surface (Figure 1c). Since there are always some Mg floating on the surface during growth because of segregation [26], the interruption will drive the floating Mg to incorporate into the Al_*x*_Ga_1 *– x*_N crystal, thus greatly enhancing Mg solubility. This result confirms that the Mg incorporation on the growing surface can be transiently enhanced further by an extremely N-rich condition interruption, thereby increasing the *C*_Mg_ that would reside at the interrupting region.

**Figure 3:**
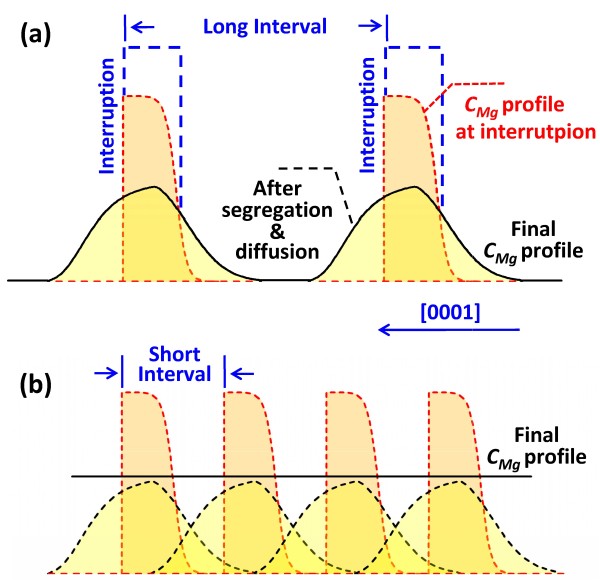
**Schematic diagram of the Mg incorporation behavior in the AlGaN grown by the MSE technique.** As the interruption interval is long, only some peaks distribute locally at the interruptions after Mg segregation and diffusion **(a)**, optimizing the interruption interval, a high and uniform Mg distribution over the entire AlGaN epilayer could be achieved **(b)**.

However, the *C*_Mg_ enhancement at the interruption region is much smaller than that on the final epilayer surface (Figure 1c), and the *C*_Mg_ far from the interruption region remains low. This result is caused by the wide interval between consecutive interruptions, considerably decreasing the *C*_Mg_ at the interruption regions and resulting in the non-uniformity of the *C*_Mg_ distribution by Mg segregation and diffusion after interruption (Figure 3a). Therefore, the interruption interval, interruption time, and growth rate should play critical roles in affecting the *C*_Mg_ overlap. As illustrated in Figure 3b, we further proposed the MSE technique, optimizing the interruption conditions, to incorporate surface Mg atoms before they can re-segregate to the surface, thus further increasing the average Mg incorporation and approaching a uniform Mg distribution over the entire AlGaN epilayer instead of being distributed locally.

**Figure 4:**
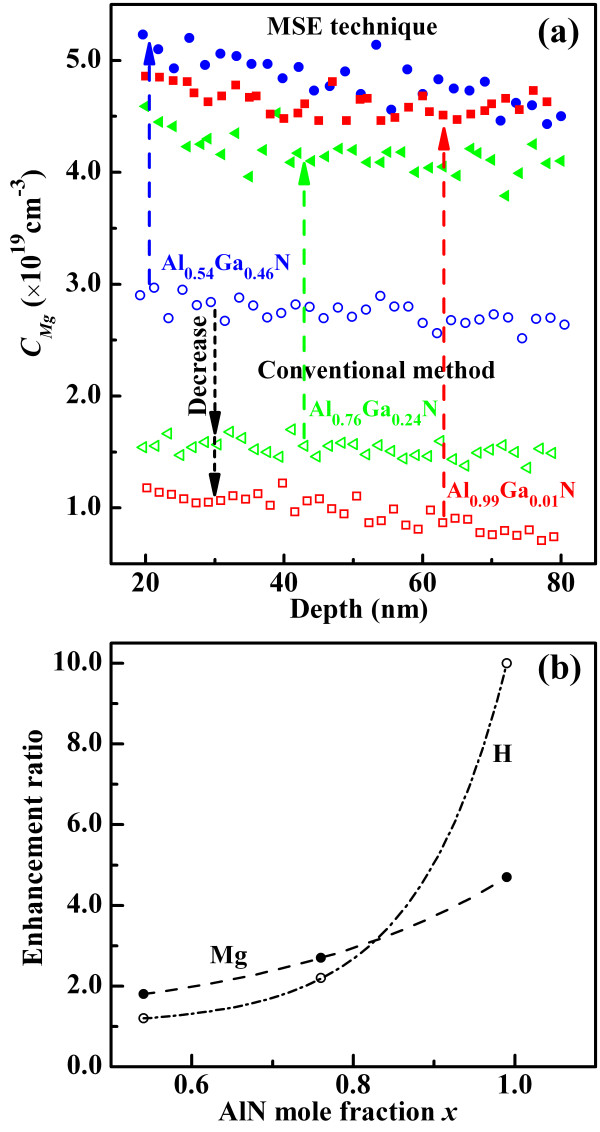
**Bulk *C***_**Mg**_** of the samples and enhancement ratios of Mg/H concentrations. (a)** Bulk *C*_Mg_ of the samples with different Al contents grown by the MSE technique and the conventional method. **(b)** Enhancement ratios of Mg and H concentrations by the MSE technique as a function of Al content compared with that of the conventional method.

Three Mg-doped Al_*x*_Ga_1 *– x*_N (*x* = 0.54, 0.76, 0.99) samples were grown by using the MSE technique (the inset of Figure 2b). An optimized 2-nm interruption interval combining with 2-s interruption time were used for all samples, with Cp_2_Mg flux of 0.81 nmol/min. As shown in Figure 4a, the samples with different Al contents exhibit high *C*_Mg_ range from 4 × 10^19^ cm^*-*3^ to 5 × 10^19^ cm^*-*3^ and homogeneous distribution at a wide region as expected, whereas the *C*_Mg_ of the samples grown via conventional method decrease with increasing Al content, which is consistent with the theoretical prediction. By comparison, the average *C*_Mg_ in the samples with different Al contents increase several times, and the enhancement ratios increase as the Al content increases, as shown in Figure 4b. Particularly, the enhancement ratio is approximately up to 5 in the Al_0.99_Ga_0.01_N. These results indicate that a high *C*_Mg_ can be easily achieved in Al-rich AlGaN by combining the surface effect with the N-rich growth atmosphere modulation.

High Mg doping was reported to result in Mg-rich precipitates. The primary Mg-rich precipitates were presumed to be Mg_3_N_2_[27,28], which can be formed when Mg do not incorporate as acceptors in the desired substitutional sites. The substitutional Mg was suggested to be usually passivated by H during growth, and the corresponding Mg acceptor can be activated by postgrowth thermal annealing to dissociate the Mg - H complex [29]. The correlation between the substitutional Mg and H was verified by previous theoretical and experimental investigations [30,31]. Thus, the H concentration is most likely associated with *C*_Mg_ if Mg is effectively incorporated in the desired substitutional sites. The enhancement ratios of H concentration for the MSE technique increase from 1.2 to 10 with increasing Al content, compared with that of the conventional method, as shown in Figure 4b. This simultaneous enhancement in H concentration demonstrates that the Mg was effectively incorporated in the desired substitutional sites by the MSE technique. In this work, the high *C*_Mg_ is the important basis for improving the hole concentration in *p*-type AlGaN epilayer. Besides the solubility limit, the high activation energy of Mg acceptors is another contribution for the low *p*-type doping of Al_*x*_Ga_1 *– x*_N, leading to a low acceptor activation probability [5,8]. In order to increase the overall *p*-type doping, more efforts on activating the obtained high *C*_Mg_ will be included in future progress.

## Conclusions

The MSE technique, which utilizes periodical interruptions under an extremely N-rich atmosphere, was proposed to enhance Mg incorporation, base on the first-principles total energy calculations. During the interruption, metal flows were closed to produce an ultimate V/III ratio condition without affecting the AlGaN growth. By optimizing the interruption conditions, we obtained a high concentration and uniform distribution Mg in the AlGaN epilayer. The *C*_Mg_ enhancements increase with increasing Al content through this method. Particularly, for the Al_0.99_Ga_0.01_N, the enhancement ratio can be achieved up to about 5 and the final Mg concentration was determined to be 5 × 10^19^ cm^–3^. Meanwhile, the simultaneous increase of the H concentration confirms the Mg effective incorporation in the desired substitutional sites instead of forming Mg_3_N_2_. The proposed approach, which is convenient as well as effective, could be used as a general strategy to promote dopant incorporation in wide bandgap semiconductors with stringent dopant solubility limits.

## Competing interests

The authors declare that they have no competing interests.

## Authors’ contributions

TCZ carried out the experiments and drafted the manuscript. WHY, WJ and HYC helped in the preparation and characterization of the samples. JCL and SPL took part in the data analysis. WL, DJC and JYK conceived the study and participated in the data analysis and the critical review of the manuscript. All authors read and approved the final manuscript.
